# Supplementary material for: Different outcomes of endurance and resistance exercise in skeletal muscles of Oculopharyngeal muscular dystrophy
Source: J Cachexia Sarcopenia Muscle. 2024 Aug 7;15(5):1976–88. doi: 10.1002/jcsm.13546 (PMC11446690; doi:10.1002/jcsm.13546)
Supplement: Supplementary file 1 — Data S1. Supporting Information. [file JCSM-15-1976-s002.docx]

**Supplemental references**

S1. Calado A, Tomé FM, Brais B, Rouleau GA, Kühn U, Wahle E *et al.* Nuclear inclusions in oculopharyngeal muscular dystrophy consist of poly(A) binding protein 2 aggregates which sequester poly(A) RNA. *Hum Mol Genet* 2000;**9**:2321–2328.

S2. Joanne P, Hourdé C, Ochala J, Caudéran Y, Medja F, Vignaud A *et al.* Impaired adaptive response to mechanical overloading in dystrophic skeletal muscle. *PLoS One* 2012;**7**:e35346.

S3. Davies JE, Wang L, Garcia-Oroz L, Cook LJ, Vacher C, O’Donovan DG *et al.* Doxycycline attenuates and delays toxicity of the oculopharyngeal muscular dystrophy mutation in transgenic mice. *Nat Med* 2005;**11**:672–677.

S4. Hourdé C, Joanne P, Medja F, Mougenot N, Jacquet A, Mouisel E *et al.* Voluntary physical activity protects from susceptibility to skeletal muscle contraction-induced injury but worsens heart function in mdx mice. *Am J Pathol* 2013;**182**:1509–1518.

S5. Mayeuf-Louchart A, Hardy D, Thorel Q, Roux P, Gueniot L, Briand D *et al.* MuscleJ: a high-content analysis method to study skeletal muscle with a new Fiji tool. *Skelet Muscle* 2018;**8**:25.

S6. Joanne P, Hovhannisyan Y, Bencze M, Daher M-T, Parlakian A, Toutirais G *et al.* Absence of Desmin Results in Impaired Adaptive Response to Mechanical Overloading of Skeletal Muscle. *Front Cell Dev Biol* 2021;**9**:662133.

S7. Roy RR, Edgerton VR. Response of mouse plantaris muscle to functional overload: comparison with rat and cat. *Comp Biochem Physiol A Physiol* 1995;**111**:569–575.

S8. Honda M, Tsuchimochi H, Hitachi K, Ohno S. Transcriptional cofactor Vgll2 is required for functional adaptations of skeletal muscle induced by chronic overload. *J Cell Physiol* 2019;**234**:15809–15824.

S9. Jorgenson KW, Phillips SM, Hornberger TA. Identifying the Structural Adaptations that Drive the Mechanical Load-Induced Growth of Skeletal Muscle: A Scoping Review. *Cells* 2020;**9**:1658.

S10. Højfeldt G, Sorenson T, Gonzales A, Kjaer M, Andersen JL, Mackey AL. Fusion of myofibre branches is a physiological feature of healthy human skeletal muscle regeneration. *Skelet Muscle* 2023;**13**:13.

S11. Cordeiro AV, Brícola RS, Braga RR, Lenhare L, Silva VRR, Anaruma CP *et al.* Aerobic Exercise Training Induces the Mitonuclear Imbalance and UPRmt in the Skeletal Muscle of Aged Mice. *The Journals of Gerontology: Series A* 2020;**75**:2258–2261.

S12. Garcia-Valles R, Gomez-Cabrera MC, Rodriguez-Mañas L, Garcia-Garcia FJ, Diaz A, Noguera I *et al.* Life-long spontaneous exercise does not prolong lifespan but improves health span in mice. *Longevity & Healthspan* 2013;**2**:14.
